# Targeted detection of cancer at the cellular level during biopsy by near-infrared confocal laser endomicroscopy

**DOI:** 10.1038/s41467-022-30265-z

**Published:** 2022-05-17

**Authors:** Gregory T. Kennedy, Feredun S. Azari, Elizabeth Bernstein, Bilal Nadeem, Ashley Chang, Alix Segil, Sean Carlin, Neil T. Sullivan, Emmanuel Encarnado, Charuhas Desphande, Sumith Kularatne, Pravin Gagare, Mini Thomas, John C. Kucharczuk, Gaetan Christien, Francois Lacombe, Kaela Leonard, Philip S. Low, Aline Criton, Sunil Singhal

**Affiliations:** 1 Department of Surgery, University of Pennsylvania School of Medicine, Philadelphia, PA USA; 2 Department of Radiology, University of Pennsylvania School of Medicine, Philadelphia, PA USA; 3 Department of Pathology, University of Pennsylvania School of Medicine, Philadelphia, PA USA; 4 On Target Laboratories, West Lafayette, IN USA; 5 Mauna Kea Technologies, Paris, France; 6 Department of Chemistry, Purdue University, West Lafayette, IN USA

**Keywords:** Non-small-cell lung cancer, Applied optics, Cancer imaging

## Abstract

Suspicious nodules detected by radiography are often investigated by biopsy, but the diagnostic yield of biopsies of small nodules is poor. Here we report a method—NIR-nCLE—to detect cancer at the cellular level in real-time during biopsy. This technology integrates a cancer-targeted near-infrared (NIR) tracer with a needle-based confocal laser endomicroscopy (nCLE) system modified to detect NIR signal. We develop and test NIR-nCLE in preclinical models of pulmonary nodule biopsy including human specimens. We find that the technology has the resolution to identify a single cancer cell among normal fibroblast cells when co-cultured at a ratio of 1:1000, and can detect cancer cells in human tumors less than 2 cm in diameter. The NIR-nCLE technology rapidly delivers images that permit accurate discrimination between tumor and normal tissue by non-experts. This proof-of-concept study analyzes pulmonary nodules as a test case, but the results may be generalizable to other malignancies.

## Abstract (web summary)

Radiography identifies suspicious lung nodules that are not always easy to diagnose via biopsy. Here, the authors utilize a fluorescent dye that targets the folate receptor and show using needle based endomicroscopy that it can be used to identify cancer cells during biopsy procedures

## Introduction

The widespread use of computed tomography scanning in medical practice and as part of expanded cancer screening guidelines has made it commonplace to detect nodules throughout the body that are radiographically suspicious for malignancy^1–4^. Care pathways for these nodules often involve tissue biopsy to rule out malignancy^5^. Current medical technology does not provide real-time diagnostic information during biopsy, instead requiring histopathologic analysis that can take several days to complete^6^. For small, difficult-to-access, or sub-solid nodules in particular, the diagnostic yield of biopsies is low, leading to patient anxiety and increased healthcare costs due to additional procedures and radiographic surveillance^7–9^.

Due to this growing clinical challenge, a number of techniques have been proposed to achieve real-time in vivo optical detection of cancer at the cellular level and thereby improve the diagnostic accuracy of biopsy-based diagnosis and staging for small nodules. Existing technologies in preclinical testing—including Raman spectroscopy, reverse contrast optical endomicroscopy, and fluorescence-lifetime imaging microscopy—require analytic time not conducive to real-time intraprocedural decision making, cannot be reliably interpreted by non-experts, or do not offer resolution at the cellular level^10–13^.

Our group and others have achieved macroscopic differentiation between tumor and normal tissue during resection of solid tumors through the use of intraoperative molecular imaging (IMI) with tumor-targeted optical tracers that are systemically administered prior to surgery^14,15^. The tracer pafolacianine consists of a NIR fluorescent dye (S0456) conjugated to a folate analog, which specifically targets malignant cells overexpressing folate receptor alpha^16^. IMI with pafolacianine has shown efficacy in discrimination of tumor tissue from normal tissue during resection of solid tumors, but its ability to localize cancer at the cellular level has not been evaluated.

Fiber-based CLE is an adaptation of conventional microendoscopy technology in which a low-power laser illuminates a tissue of interest and transmits light reflected from the tissue via a flexible optical fiber bundle to generate high-resolution tissue images^17^. Both probe-based CLE (pCLE) and needle-based CLE (nCLE) permit autofluorescence imaging as well as reverse-contrast imaging of tissues after systemic administration of fluorescein^11,13,18,19^. Recent studies conducted by Annema and colleagues have shown the benefit of fluorescein-based nCLE as a diagnostic adjunct in the bronchoscopic diagnosis and staging of lung cancer^13,19^.

**Fig. 1:**
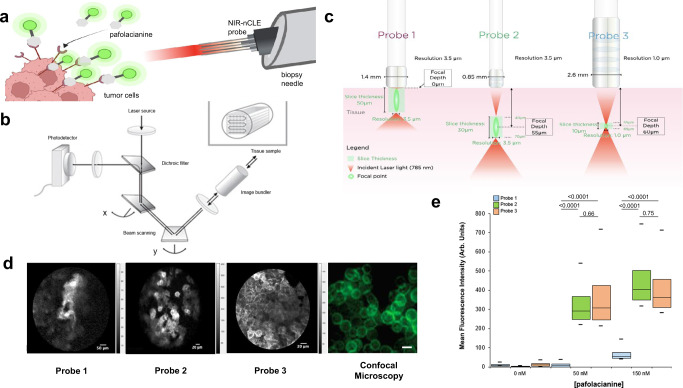
NIR-nCLE detects pafolacianine-labeled cells in culture. **a** Schematic overview of the principle behind NIR-nCLE: the detection of individual malignant cells labeled with a targeted NIR tracer within the lumen of a biopsy needle. **b** Technical overview of the three NIR-nCLE probes tested in the study. **c** Schematic representation of the technology underlying CLE. A laser beam is directed to the tissue sample via an image bundler. The resulting emission of light is retrieved and filtered through a pinhole before reaching the photodetector. x and y represent the two axes of the CLE image. **d** Representative images taken with the three NIR-nCLE probes of pafolacianine-labeled KB cells in culture and compared to images obtained with conventional confocal microscopy (*n* = at least 50 imaging frames from three independent samples). Scale bars represent 20 µm. **e** Mean fluorescence intensity (MFI) of KB cells stained with increasing concentrations of pafolacianine and imaged with the three NIR-nCLE probes. Minima, center, and maxima of boxes represent 25th percentile, mean, and 75th percentile of MFI obtained for the entire imaging sequence. (at 0 nM pafolacianine, *n* = 88, 100, and 96 frames for Probes 1, 2, and 3, respectively; at 50 nM pafolacianine, *n* = 88, 188, and 123 frames for Probes 1, 2, and 3, respectively; at 150 nM pafolacianine, *n* = 71, 350, and 66 frames for Probes 1, 2, and 3, respectively). Whiskers represent the 5th and 95th percentile of MFI over the same sequence. Statistical comparisons were performed using two-sided *t* tests and *p* values are shown on the graph.

In this work, we describe a method of integrating these two technologies to achieve real-time detection of cancer at the single cell level during biopsy. This method—which we term near-infrared needle-based confocal laser endomicroscopy (NIR-nCLE)—combines [i] near infrared imaging using pafolacianine with [ii] an ultra-thin, flexible nCLE system to exploit both the cancer-specificity of the targeted NIR tracer as well as the cellular-level resolution of CLE (Fig. 1a). As a proof-of-concept, we evaluated the technology for identification of malignant cells during biopsy of solitary pulmonary nodules (SPNs), which are the most common incidental nodule detected annually in the United States^20^. Using a number of preclinical models including surgically resected human ground glass opacity (GGO) pulmonary nodules, we found that the NIR-nCLE achieved rapid, sensitive detection of cancer cells during biopsy procedures with high diagnostic accuracy when compared to formal histopathologic analysis.

## Results

### Development and testing of NIR-nCLE probes

Three flexible, ultra-thin NIR-nCLE probes were developed (Fig. 1b). Probe 1 contains 30,000 optical fiber bundles with an imaging depth of 0–50 μm, a lateral resolution of 3.5 μm and a field of view of 600 μm. Probe 2 contains 10,000 optical fiber bundles with an imaging depth of 55 μm, a lateral resolution of 3.5 μm and a field of view of 325 μm. Probe 3 contains 30,000 optical fiber bundles with imaging depth of 60μm, a lateral resolution of 1 μm and a field of view of 240 μm.

The probes were used in conjunction with a commercially available CLE system (CellVizio, Mauna Kea Technologies, Paris, France) that generates an excitation light with a 785 nm laser which is expanded by a beam expander and reflected by a dichroic mirror (Fig. 1c). Subsequently, a two-dimensional scanning system scans the beam in the two-dimensional plane. After the scanning, the beam is relayed into the back aperture of the objective lens, which couples the excitation light into the proximal end of the optical fiber bundle in the NIR-nCLE probe. Finally, the beam is focused on the sample by a near-infrared miniature objective (at the imaging depth), which also collects the emitted fluorescence from the sample. The fluorescence at longer wavelengths is transmitted along the same path and passed through the dichroic mirror and a bandpass filter (800–890 nm). After passing the bandpass filter, the fluorescence is focused by a lens to pass through a pinhole that is placed at the focus of the condenser. The pinhole is used to reject the out-of-focus light. Finally, a photodiode is used to acquire the optical signals and convert them into electrical signals. With the X-Y scanners, the imaging speed can reach 8–12 frames per second, which makes imaging in real-time possible. Each frame consists of 512 × 512 pixels for the Probes 1 and 3 and 320 × 322 pixels for Probe 2.

### NIR-nCLE detects NIR signal from pafolacianine labeled cells

To determine whether the CLE probes could detect pafolacianine-labeled cells in culture, KB cells, a human cervical carcinoma cell line with known overexpression of FRα, were stained with varying concentrations of pafolacianine. KB cells have previously been used as a positive control for in vitro and in vivo evaluation of pafolacianine-based imaging, and were therefore used as the initial cell line examined in our study^21^. Subsequent experiments were conducted with primary lung cancer cell lines. The stained cells were imaged with the three NIR-nCLE probes (Fig. 1c, d). Probes 2 and 3 were able to detect malignant cells when stained with 50 nM pafolacianine with mean fluorescence intensity (MFI) of tumor cells of 368.2 arbitrary units (arb. units, IQR: 292.6–539.8 arb. units) and 426.1 arb. units (IQR: 308.2–717.6 arb. units), respectively. Probe 1 was not able to detect malignant cells in culture until concentrations of 150 nM pafolacianine. Cancer cell MFI by probe 1 was 75.4 arb. units (IQR: 55.3–144.9 arb. units), which was significantly lower than MFIs by probe 2 or probe 3 (MFI = 503.2 arb. units [IQR: 404.0–743.7 arb. units] for probe 2, MFI = 457.4 arb. units [IQR:363.2–711.5 arb. units] for probe 3, *p* < 0.0001 in both cases). Given the equivalent performance of probes 2 and 3 in detecting malignant cells in culture as well as its smaller size, probe 2 was chosen for further testing.

### NIR-nCLE discriminates cancer from normal cells at the single-cell level in culture

**Fig. 2:**
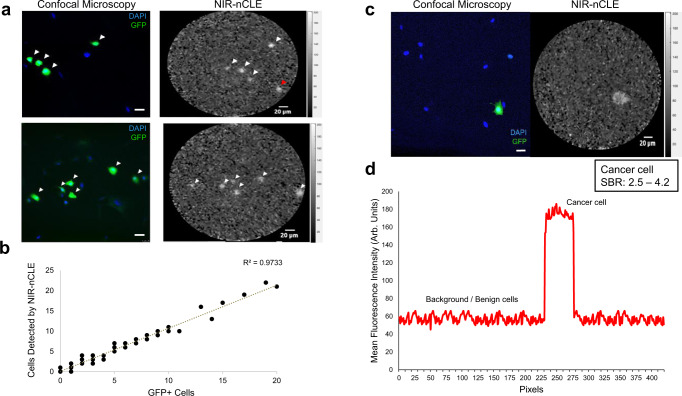
NIR-nCLE discriminates malignant cells from normal cells in culture. **a** Paired confocal microscopy and NIR-nCLE images of GFP-transfected lung adenocarcinoma (A549-GFP) cells co-cultured with normal lung fibroblast (HD28) cells. White arrowheads indicate NIR-nCLE fluorescence concordant with confocal microscopy at GFP wavelength and red arrowheads indicate discordant fluorescence. Scale bars represent 20 µm. **b** Correlation of the number of cancer cells detected by NIR-nCLE in individual still images and compared to the number detected by paired confocal microscopy images at GFP wavelength as the gold standard. **c** Detection of a single malignant cell by NIR-nCLE when A549-GFP and HD28 cells were co-cultured at a ratio of 1:1,000. Scale bars represent 20 µm. **d** Fluorescence intensity cross-sectional profile of a NIR-nCLE image of a single malignant cell, illustrating the high SBR (range 2.5–4.2) of cancer cells in culture imaged by NIR-nCLE.

To determine the ability of NIR-nCLE to differentiate malignant from benign cells, GFP-transfected human pulmonary adenocarcinoma (A549-GFP) cells were co-cultured with normal lung fibroblast (HD28) cells at various ratios (1:1, 1:10, 1:100, 1:1000, 1:10,000). Cells were stained with 50 nM pafolacianine and imaged with NIR-nCLE after 2 h (Supplementary Fig. 1). Images were then compared to conventional confocal microscopy at GFP wavelength (Fig. 2a). There was high concordance between NIR-nCLE fluorescence and GFP fluorescence (Fig. 2b, *R*^2^ = 0.9733) with slight overestimation of malignant cell number by NIR-nCLE (trendline: *y* = 1.0634x + 0.0571). The lower limit of detection of a single malignant cell per high powered field was when A549-GFP cells and HD28 cells were co-cultured at a ratio of 1:1000 (Fig. 2c). Individual cancer cells displayed distinctly higher fluorescence intensity than normal background cells with signal-to-background ratio (SBR) ranging from 2.5 to 4.2 (Fig. 2d).

### NIR-nCLE differentiates malignant cells from normal lung parenchyma in an orthotopic murine model

**Fig. 3:**
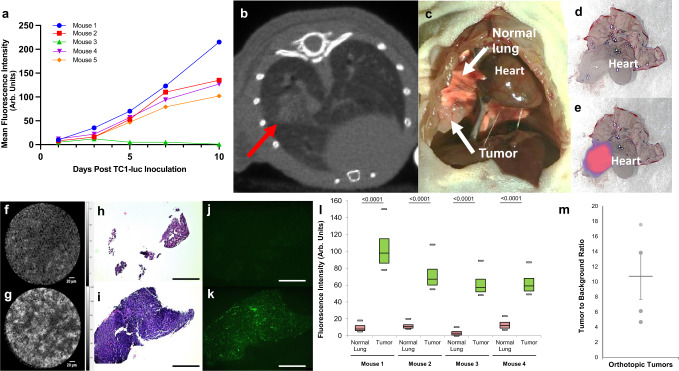
NIR-nCLE distinguishes tumor from normal lung in an orthotopic murine model. **a** Growth of orthotopic murine lung adenocarcinoma (TC1-luc) tumors as assessed using bioluminescence imaging with a NIR luciferin analog. **b** Respiratory gated CT image of a representative tumor at 10 days, with red arrow marking the tumor. **c**, **d** White light images of an orthotopic tumor in situ and ex vivo 24 h after infusion with pafolacianine (2 mg/kg). Animals were killed prior to thoracic cavity exposure, organ harvest, and image acquisition. **e** NIR image of the orthotopic tumor showing clear macroscopic fluorescent signal. **f**, **g** Representative NIR-nCLE images of normal and tumor tissue, respectively. **h**, **i** Hematoxylin and eosin-stained slides of biopsies taken from the site of NIR-nCLE imaging. Scale bars represent 5 mm. **j**, **k** Fluorescence microscopy of biopsy slides from normal lung and tumor tissue, respectively. Scale bars represent 5 mm. **l** Mean fluorescence intensity (MFI) of NIR-nCLE sequences taken during biopsy of normal lung and tumor tissue for each mouse. Minima, center, and maxima of boxes represent 25th percentile, mean, and 75th percentile of MFI obtained for the entire imaging sequence. (*n* = 117, 117, 117, and 118 frames for normal lung biopsies for Mice 1, 2, 3, and 4, respectively; *n* = 116, 111, 110. And 117 frames for tumor biopsies for Mice 1, 2, 3, and 4, respectively). Whiskers represent the 5th and 95th percentile of MFI over the same sequence. Statistical comparisons were performed using two-sided *t* tests and *p* values are shown on the graph. **m** Tumor-to-background ratios of the orthotopic TC1-luc tumors (*n* = 4 biologically independent animals). Each tumor is represented by a single point, with mean line and standard error bars also shown.

To determine whether NIR-nCLE can detect malignant cells in vivo, mice bearing luciferase transfected pulmonary adenocarcinoma (TC1-luc) tumors (*n* = 4) were administered pafolacianine (2 mg/kg) and lungs were harvested *en bloc* 24 h after tracer injection (Fig. 3). NIR-nCLE and biopsy of tumor and normal lung were performed within the lumen of a 19 G biopsy needle. MFI of NIR-nCLE for tumor tissue was 75.0 arb. units (IQR: 62.0–80.3 arb. units) and 9.6 arb. units (IQR: 8.1–12.2 arb. units) for normal lung (*p* < 0.0001). NIR-nCLE fluorescence was concordant with fluorescence detected on immunofluorescence microscopy in all biopsy specimens. Mean TBR for orthotopic TC1-luc tumors was 10.8 (TBR range: 4.7–17.5). As a negative control, mice bearing orthotopic TC1-luc tumors (*n* = 3) were administered PBS vehicle and lungs were harvested *en bloc* 24 h after tracer injection. NIR-nCLE and tissue biopsies were performed as above, and there were no significant differences between MFI for normal lung and tumor tissue (Supplementary Fig. 2, mean MFI for tumor = 3.6 arb. units vs 2.4 arb. units for normal lung, *p* = 0.22).

### NIR-nCLE can diagnose malignancy during transthoracic and transbronchial biopsy of pulmonary tumors in small animal models

**Fig. 4:**
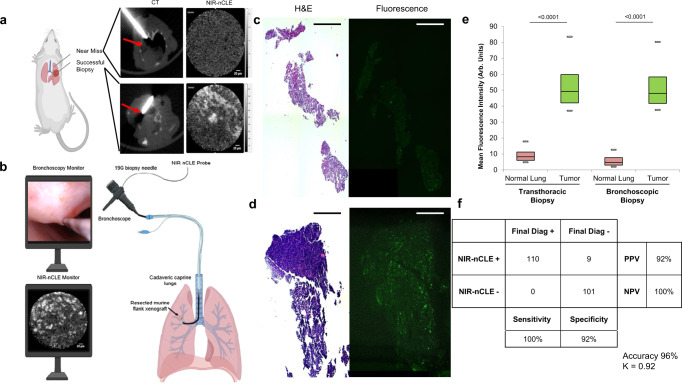
NIR-nCLE can diagnoses malignancy in real-time during transthoracic and bronchoscopic biopsy of pulmonary tumors in small animal models. **a** Overview of the transthoracic biopsy model. Nude, athymic mice bearing orthotopic human non-small cell lung cancer xenografts (*n* = 8) underwent CT-guided NIR-nCLE and biopsy. Representative CT and NIR-nCLE images of a near-miss and successful biopsy are shown with red arrow marking the tumor location. **b** Overview of the bronchoscopic biopsy model. Cadaveric caprine lungs (*n* = 3) were implanted with murine flank human non-small cell lung cancer xenografts resected under pafolacianine guidance. Tumors were localized using a standard therapeutic bronchoscope and NIR-nCLE and biopsy was conducted via the working port of the bronchoscope using a 19G flexible biopsy needle. Representative bronchoscopic and NIR-nCLE images during tumor biopsy are shown. **c**, **d** Representative hematoxylin and eosin stained biopsy slides and paired fluorescence microscopy of normal lung and tumor, respectively. Scale bars represent 5 mm. **e** Mean fluorescence intensity (MFI) of NIR-nCLE sequences taken during biopsy of normal lung and tumor tissue in the transthoracic (*n* = 10 biologically independent animals) and bronchoscopic (*n* = 3 biologically independent animals) biopsy models. Minima, center, and maxima of boxes represent 25th percentile, mean, and 75th percentile of MFI obtained for the entire imaging sequence. (for transthoracic biopsies *n* = 922 and 1408 frames for normal lung and tumor biopsy, respectively; for bronchoscopic biopsies *n* = 423 and 471 frames for normal lung and tumor biopsy, respectively). Whiskers represent the 5th and 95th percentile of MFI over the same sequence. Statistical comparisons were performed using two-sided *t* tests and *p* values are shown on the graph. **f** Diagnostic performance of NIR-nCLE sequences when analyzed by 5 non-expert raters, blinded to the final histopathologic diagnosis associated with each sequence.

To determine whether NIR-nCLE can differentiate tumor cells from normal lung tissue during biopsy, we developed animal models of both transthoracic needle biopsy (Supplementary Fig. 3) and bronchoscopic needle biopsy (Supplementary Fig. 4). In the transthoracic biopsy model, mice (*n* = 8) bearing human non-small cell lung cancer (A549 or H1264) orthotopic xenografts underwent CT-guided NIR-nCLE and biopsy of tumor and normal lung 24 h after pafolacianine administration (Fig. 4a). In the bronchoscopic biopsy model, we implanted caprine lungs (*n* = 3) with A549 flank xenografts harvested from nude athymic mice that had been administered pafolacianine prior to resection. We subsequently intubated the lungs with a clinical grade bronchoscope and took NIR-nCLE and biopsies of tumor and normal lung using a flexible 19G biopsy needle (Fig. 4b).

In both models, NIR-nCLE fluorescence was concordant with malignancy as assessed by hematoxylin and eosin staining and immunofluorescence microscopy (Fig. 4c, d). In the transthoracic biopsy model, MFI of NIR-nCLE was 49.2 arb. units (IQR: 42.0–59.8 arb. units) for tumor tissue and 8.2 arb. units (IQR: 6.0–11.2 arb. units) for normal lung (Fig. 4e, *p* < 0.0001). In the bronchoscopic biopsy model, MFI of NIR-nCLE was 48.0 arb. units (IQR: 41.7–58.3 arb. units) for tumor tissue and 6.7 arb. units (IQR: 5.0–9.7 arb. units) for normal lung (*p* < 0.0001). SBR for tumor biopsies ranged between 3.4 and 10.4.

To evaluate the diagnostic performance of NIR-nCLE, all imaging sequences (*n* = 44) captured in the biopsy models were randomized. There were 22 sequences corresponding to malignant tissue biopsies and 22 sequences corresponding to non-malignant tissue biopsies. After a brief training session introducing the concept of NIR-nCLE and representative examples of malignant and non-malignant biopsy sequences(<5 min), five blinded reviewers independently assigned a diagnosis for each NIR-nCLE sequence based on the presence of fluorescent cells. Blinded raters had no role in the acquisition or analysis of NIR-nCLE sequences. The blinded raters scored the NIR-nCLE videos with an overall sensitivity and specificity of 100% and 92%, respectively (Fig. 4f). Importantly, there were no false negative diagnoses among the 220 individual ratings. The overall inter-observer agreement of the five raters was excellent (*κ* = 0.92).

### NIR-nCLE accurately discriminates tumor from normal tissue in human resection specimens

**Fig. 5:**
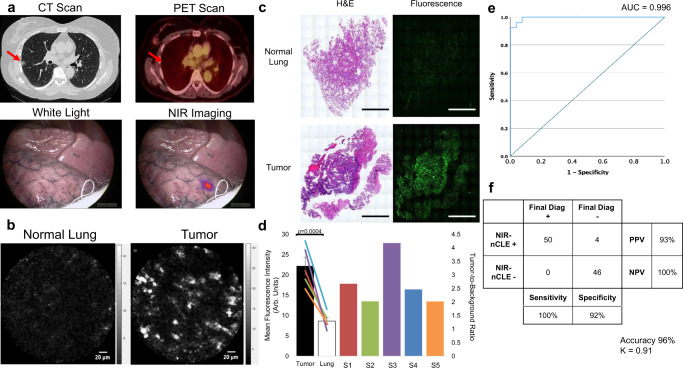
NIR-nCLE can discriminate tumor from normal lung in sub-solid human tumors. **a** Preoperative and intraoperative imaging from a representative patient with a small ground glass opacity (GGO, marked by red arrow) that was preoperatively infused with pafolacianine. The lesion is not identifiable by white light imaging but is clearly labeled on NIR imaging. **b** NIR-nCLE images of normal lung and tumor taken on ex vivo specimens. **c** H&E and fluorescence microscopy of biopsy specimens taken at the site of NIR-nCLE imaging. Scale bars represent 5 mm. **d** Fluorescence analysis of NIR-nCLE sequences. Black and white bars represent the MFI of tumor and normal lung biopsy sequences, respectively, averaged across all biopsy specimens (*n* = 10 tumor biopsies from biologically independent samples and *n* = 10 normal lung biopsies from biologically independent samples). Data are presented as mean values ± SEM. Colored lines represent the MFI of tumor and normal lung for individual study participants. The colored bars represent the tumor-to-background ratios of individual subjects and colors correspond to those of lines depicting MFI. Statistical comparisons were performed using two-sided *t* tests and *p* values are shown on the graph. **e** Receiver operating characteristic (ROC) analysis showed that high area under the curve (AUC) was obtained for MFI (AUC = 0.996, 95% CI: 0.986–1.000). **f** Diagnostic performance of NIR-nCLE sequences when analyzed by 5 non-expert raters, blinded to the final histopathologic diagnosis associated with each sequence.

To determine whether NIR-nCLE can differentiate tumor from normal lung in human tumors, we conducted NIR-nCLE guided biopsy of tumor and normal lung tissue in resection specimens of five patients undergoing pafolacianine-guided lung cancer resection as part of an ongoing Phase 1 clinical trial (Fig. 5 and Supplementary Fig. 5). Patients included in the study tended to be female (*n* = 4, 80%), middle-aged (mean age: 67.4 years), and former smokers (15.5 mean pack years). All nodules imaged in this study were ground glass opacities (GGOs). GGOs are a subset of SPNs that are often early-stage adenocarcinoma spectrum lesions. They have a soft tissue architecture and are particularly challenging to localize and diagnose via existing bronchoscopic technology^22^. Mean lesion size was 1.9 cm. By final histopathologic analysis, the resected lesions were mostly invasive adenocarcinomas (*n* = 3, 60%) with one minimally invasive adenocarcinoma and one adenocarcinoma in situ. Mean MFI by NIR-nCLE for tumor biopsy was 22.1arb. units and mean MFI for normal lung was 8.56 arb. units (*p* < 0.0001). Mean TBR for pulmonary tumors was 2.7 (range: 2.0–4.2). Receiver operating characteristic (ROC) analysis showed that a high area under the curve (AUC) was obtained for MFI (AUC = 0.996, Fig. 5e).

To evaluate the diagnostic performance of NIR-nCLE in human tumors, all imaging sequences captured in the human study (*n* = 20) were randomized and assigned a diagnosis by five blinded reviewers that were not involved in the acquisition of NIR-nCLE sequences (Fig. 5f). There were 10 sequences corresponding to malignant tissue biopsies and 10 sequences corresponding to non-malignant tissue biopsies. Blinded raters assessed NIR-nCLE videos with an overall sensitivity and specificity of 100% and 92%, respectively. Again, there were no false negative diagnoses among the 100 individual ratings. Overall diagnostic accuracy was 96% and intra-observer agreement was excellent (*κ* = 0.91).

## Discussion

In summary, we have established the feasibility of a method to detect cancer at the cellular level in real-time during biopsy of nodules suspicious for malignancy. This technology, which we term NIR-nCLE, combines NIR imaging using a cancer-targeted tracer with a needle-based CLE system modified to detect optical signal in the NIR spectrum. We found that the technology has the resolution to identify a single cancer cell among normal fibroblast cells when co-cultured at a ratio of 1:1000, and can identify small volumes of disease in human pulmonary sub-solid nodules <2 cm in diameter. Furthermore, the technology delivers easily interpretable images in real-time that permit highly accurate discrimination between tumor and normal tissue by non-expert observers. Our study specifically analyzed pulmonary nodules as a test case, but the results are likely generalizable to other malignancies and offer great promise to improve the diagnostic yield of biopsy-based diagnosis and staging of small nodules.

NIR-nCLE represents the synergistic integration of two existing technologies, and thereby substantially advances both tumor-targeted NIR imaging and optical endomicroscopy. Targeted NIR imaging of tumors has been extensively studied as an adjunct to surgeons’ ability to differentiate tumor from normal surrounding tissue during cancer resection^23,24^. Existing NIR imaging modalities can identify tumor at the macroscopic level, but this study demonstrates microscopic NIR identification of cancer cells in vivo. CLE has been previously studied for endoscopic evaluation of nodules in a variety of organ systems^19,25–27^. Prior work in nCLE imaging has relied upon tissue autofluorescence or reverse-contrast imaging in the far-red spectrum after systemic administration of fluorescein^11,18^. By targeting tumor cells directly, NIR-nCLE can reliably detect very small quantities of cancer cells, and also provides images with a binary readout (presence or absence of fluorescent cells) that can be interpreted by non-experts in real-time with excellent inter-observer agreement.

We found that NIR-nCLE can detect single malignant cells when co-cultured with normal lung fibroblast cells up to a ratio of 1:1000. This is an important finding given that tumors consist of a significant amount of non-malignant cells and extracellular components that comprise the tumor microenvironment^28^. Lung cancers, in particular, contain large quantities of non-cancerous elements including tumor-associated fibrosis^29^. In a recent ex vivo study of 64 resected pulmonary nodules, the percentage of tumor-associated fibrosis varied widely among the nodules, from 0.2 to 83.9% of the tumor volume^30^. A conservative extrapolation from the in vitro sensitivity of NIR-nCLE in the present study suggests that this technology would be able to detect malignant cells even in tumors with a very high degree of fibrosis.

We evaluated NIR-nCLE in multiple preclinical models of pulmonary nodule biopsy, and demonstrated substantial efficacy of the technology to guide both transthoracic and bronchoscopic biopsy of SPNs. When evaluating the technology’s efficacy in human SPNs, we specifically selected patients with small sub-solid, ground glass opacities (GGOs). GGOs are the most challenging pulmonary lesions to evaluate during diagnostic biopsy because they are often early-stage adenocarcinoma spectrum lesions that have a soft tissue architecture not easily distinguishable from normal lung parenchyma^22^. GGOs, unlike solid nodules, cannot be seen using fluoroscopy and endobronchial ultrasound (EBUS), and no technique is available for real-time visualization of GGOs^6,31,32^. In all five GGOs evaluated in this study, fluorescent cancer cells were identified within the lesion during biopsy. The overall MFI of GGO tumor biopsies was lower than the MFI of mouse tumor biopsies in the preclinical models. We hypothesize that this was due to the heterogeneous composition of human tumors, containing both FRα-expressing cancer cells and non-cancerous cells comprising the tumor microenvironment. Murine flank xenografts primarily contain a clonal population of malignant cells overexpressing FRα, thereby accounting for the higher degree of pafolacianine uptake and overall higher MFI. Blinded, non-expert observers were able to accurately and consistently distinguish NIR-nCLE imaging sequences within the GGOs from those obtained in normal lung parenchyma, pointing to the clinical utility of this technology as an adjunct to bronchoscopic or transthoracic biopsy of GGOs.

While we specifically analyzed pulmonary nodules as a proof-of-principle study, we anticipate that NIR-nCLE may be a beneficial adjunct to biopsy-based diagnosis and staging of nodules suspicious for malignancy in other organs. Both technologies—NIR intraoperative imaging and either probe-based CLE or needle-based CLE—have independently proven to be effective in a range of malignancies, including head and neck cancer^33,34^, breast cancer^35,36^, gastric cancer^26,37^, and colorectal cancer^38,39^. Therefore, it is likely that the integration of the two technologies by NIR-nCLE may also prove useful in these cancers. We anticipate that NIR-nCLE will improve the diagnostic yields of biopsy procedures for a range of malignancies, and molecular imaging techniques will provide important adjuncts to traditional cytological evaluation and diagnosis.

This study has several important limitations. First, NIR-nCLE will need to be evaluated in vivo during biopsy procedures to assess the impact of respiratory variation, blood circulation, and motion artifact on the technology’s efficacy. Next, the use of fluorescence intensity signal to distinguish between tumor and normal tissue is inherently limited by a number of elements including focal plane position, tissue heterogeneity, receptor expression profile, depth of signal penetration, and number of labeled cells within a particular view, among other factors. Determination of a fluorescence intensity threshold that definitively identifies malignancy is the subject of ongoing study in our laboratory, and may ultimately obviate the need for subjective image interpretation by human observers. Furthermore, machine learning approaches may aid in image standardization and interpretation as the technology undergoes further iterations in the pathway to clinical translation. There were also a small number of false-positive diagnoses in both the in vitro and in vivo models. We attribute these false positive cases to the dynamic fluorescence scale of the CLE device, which adjusts the field of view to the highest fluorescent intensity value in that single field. Therefore, areas with a small absolute fluorescence intensity may appear to have a high relative fluorescence intensity if imaged in a field with overall low fluorescence intensity. As indicated above, optimization of fixed fluorescence scales and threshold values will be needed as the technology is further developed.

In this study, we used a folate receptor-targeted NIR tracer, pafolacianine, to detect malignant cells, and note that this tracer may not be effective for tumors that do not overexpress folate receptor alpha. However, we anticipate that NIR-nCLE will be able to detect a broad range of NIR tracers and the choice of tracers can be tailored to individual patients based upon the location of the nodule in question. This study suggests the clinical promise of NIR-nCLE, but further evaluation is needed in human trials of NIR-nCLE to guide biopsy of nodules suspicious for malignancy.

## Methods

### Ethical approval

All animal studies were approved by the University of Pennsylvania Institutional Animal Care and Use Committee (Protocol #803344 and #806483). Maximum tumor burden was 2 cm in diameter and mice were regularly examined to ensure that this limit was not exceeded. Supervision of animal facilities by a board certified veterinarian further ensured compliance with all ethical protocols. The study on human specimens was approved by the University of Pennsylvania Institutional Review Board (Protocol #822153) and all subjects gave written informed consent. Subjects were not compensated for their participation in the study.

### Study drug

Pafolacianine (chemical formula C_61_H_63_N_9_Na_4_O_17_S_4_; molecular weight, 1414.42 Da) is a folate analog conjugated to the NIR fluorescent dye S0456. It is now in late-stage clinical trials of fluorescence-guided surgery for a number of folate receptor-positive malignancies including non-small cell lung cancer and ovarian cancer^14,15,40,41^. The tracer targets the folate receptor alpha which is overexpressed 90% of pulmonary adenocarcinomas and 70% of squamous cell carcinomas, and has been shown to identify solitary pulmonary nodules as small as 2 mm^42–44^. Pafolacianine maximally excites at a wavelength of 774–776 nm and has a peak emission of 794–796 nm^16^.

Pafolacianine (>96% purity) was obtained via collaboration with Philip Low, PhD (Purdue University, West Lafayette, IN) and On Target Laboratories (West Lafayette, IN). Pafolacianine was synthesized and manufactured at Aptuit in compliance with Good Manufacturing Practices. Pafolacianine was stored at −20 °C in vials containing 6 mg pafolacianine free acid in 3 mL water. Before utilization, the frozen vials were thawed, vortexed, and then diluted with 0.9% NaCl, 5% dextrose or culture media.

### Cell lines

The human cervical carcinoma cell line KB has high levels of FRα expression and served as a positive control^21^. This cell line was obtained from the American Type Culture Collection (Cat # CCL-7). Human non-small cell lung cancer cell lines were obtained from the laboratory of Dr. Steven Albelda including A549 (adenocarcinoma), H1264 (squamous cell carcinoma), and A549-GFP (green-fluorescent protein transfected adenocarcinoma). The human lung fibroblast cell line HD28 and the luciferase transfected mouse adenocarcinoma cell line TC1-luc were also obtained from the laboratory of Dr. Steven Albelda. The original source of these cell lines was the American Type Culture Collection (ATCC). Cell lines were maintained in vitro using media containing RPMI, 10% fetal bovine serum (FBS), 2 mmol/L glutamine, and 5 mg/mL penicillin/ streptomycin. Cell lines were regularly tested and maintained negative for *Mycoplasma spp*.

### In vitro testing

The first experiment was conducted to evaluate the capability of CLE to detect pafolacianine-labeled cells in culture. KB cells (50,000 cells/well in 1 mL) were seeded into poly-d-lysine microwell Petri dishes (150 mm diameter × 25 mm height) and allowed cells to form monolayers over 12 h. The spent medium was replaced with fresh medium containing pafolacianine with different concentrations (0 nM, 50 nM, 150 nM), and cells were incubated for 2 h at 37 °C. After rinsing with fresh medium (2 × 1.0 mL) and PBS (1 × 1.0 mL), fluorescence images were acquired with the 3 NIR-nCLE probes and compared to conventional confocal microscopy (Leica Microsystems, Wetzlar, Germany).

To determine whether NIR-nCLE could differentiate tumor cells from normal cells, GFP-transfected A549 cells were co-cultured with normal lung fibroblast HD28 cells at varying ratios (1:1, 1:10, 1:100, 1:1000, 1:10,000). Cells were stained with 50 nM pafolacianine as above and imaged with NIR-nCLE. Representative images of each cell plate as determined by the study personnel acquiring the images were then compared to conventional confocal microscopy at GFP wavelength (510 nm). Fifty still NIR-nCLE images were analyzed and the number of fluorescent cells per field were counted. These cell counts were compared the number of GFP expressing cells in the same field as the gold standard.

### Murine orthotopic allograft model

Female, 6–8-week-old, athymic nude mice (Charles River Laboratories, Shrewsbury, MA; *n* = 5) were transpleurally injected with TC1-luc cells diluted in a 1:1 mixture of PBS and Geltrex (Sigma Aldrich, St. Louis, MO) according to methods described previously^45^. All mice were housed in the vivarium at the University of Pennsylvania, where they were maintained on a normal mouse chow diet and a 12 h/12 h light (<10 lux)/dark cycle. The temperature ranged from 75 to 76 °F, and the humidity from 30 to 40%. Tumor growth was monitored every 2 days by bioluminescence imaging after intraperitoneal injection of 100 μL of 5 mM NIR luciferin analog (TokeOni, Sigma Aldrich, St. Louis, MO)^46^. Once tumors had been established, mice were administered 2 mg/kg pafolacianine via tail vein injection. Twenty-four hours after injection, mice were euthanized, lungs were harvested, and underwent NIR-nCLE imaging via the lumen of a 19G biopsy needle. Following NIR-nCLE image acquisition, tissue biopsies were taken at the same site.

### Transthoracic needle biopsy model

Female, 6–8-week-old, athymic nude mice bearing A549 or H1264 orthotopic xenografts (*n* = 5/group) were established by transpleural inoculation as above. Tumor growth was assessed by serial respiratory-gated CT imaging (X-Cube, Molecubes, Gent, Belgium). When tumors had reached at least 25 mm^3^ by imaging, mice were systemically administered 2 mg/kg pafolacianine and killed 24 h later. Transthoracic NIR-nCLE and 19G needle biopsy of tumor and normal lung tissue was performed under guidance from scout CT images (U-CT, MILabs, Houten, The Netherlands).

### Transbronchial biopsy model

To establish a large animal model of bronchoscopic biopsy, cadaveric caprine lungs (*n* = 3) were obtained from the Animal Model Core of the New Bolton Center of University of Pennsylvania School of Veterinary Medicine. Caprine lungs were implanted with murine flank xenografts that had been resected under guidance of pafolacianine fluorescence. Using a high-resolution therapeutic bronchoscope (Seesheen Medical Technology, Zhuhai, China) and a flexible 19G biopsy needle (Cook Medical, Bloomington, IN) through the working port of the bronchoscope, transbronchial NIR-nCLE and biopsies of tumor and normal lung were taken under optical fiber-based confocal microscopy guidance.

### Pilot study of NIR-nCLE in human tumors

Pulmonary resection specimens were obtained from five patients with small (<2 cm) ground glass opacities (GGOs) that had undergone pafolacianine-guided resection of lung tumors in a Phase 1 study (NCT02602119). The NIR-nCLE study was not an interventional Phase 1 trial, as NIR-nCLE testing was not performed in humans, but rather in human resection specimens ex vivo. The study on human specimens was approved by the University of Pennsylvania Institutional Review Board. All subjects gave written informed consent. Participants received pafolacianine (intravenous, 0.025 mg/kg) 24 h prior to resection. Following resection, the specimens were visually inspected and palpated to identify the lesion of interest on the back table. NIR-nCLE imaging followed by tissue biopsy was performed at two sites within the lesion and two sites in distant, grossly normal lung tissue.

### Histopathologic and fluorescence analysis of biopsy specimens

All biopsy specimens were imaged with the Odyssey imaging system (LI-COR Biosciences, Lincoln, NE) or the Iridium imaging system (Vision Sense, New York, NY). Tissue sections were further analyzed by hematoxylin and eosin staining and fluorescence microscopy (Leica Microsystems, Wetzlar, Germany). Final histopathologic diagnosis was rendered by a board-certified thoracic pathologist.

### Blinded rater evaluation of NIR-nCLE sequences

Five raters, blinded to the histopathology diagnoses, independently scored a randomized sequence of videos for the presence of fluorescent cells in each NIR-nCLE sequence (yes/no) and their diagnosis (malignant/benign) based upon their interpretation of the sequence. There were 320 ratings in total. These ratings were subsequently compared to the final histologic diagnoses associated with each NIR-nCLE sequence. Sensitivity, specificity, positive predictive value, negative predictive value, and accuracy were calculated according to standard definitions. The inter-observer agreement (IOA) was calculated with MATLAB R2020b (MathWorks, Natick, Massachusetts, USA) using multi-rater Fleiss’ κ^47^. The results of the IOA were interpreted according to the Landis-Koch interpretation system: poor <0.2, fair 0.21–0.4, moderate 0.41–0.6, substantial 0.61–0.8, and excellent 0.81–1^48^.

### Quantification of fluorescence in NIR-nCLE sequences

The mean fluorescence of each of the entire CLE imaging sequences was calculated using MATLAB R2021a (MathWorks, Natick, Massachusetts, USA) with dedicated software that finds clusters in histograms allowing the segmentation in NIR-nCLE frames. The average signals in the fluorescent and background areas and standard deviation for each acquisition were calculated. The average tumor fluorescent signal from each NIR-nCLE sequence of the tumor was normalized by the average fluorescent signal from each NIR-nCLE sequence of the normal lung from the same subject by dividing the average tumor fluorescent signal by the average normal tissue fluorescent signal (background).

### Statistics and reproducibility

Statistical comparison of fluorescence intensity values was conducted by paired student’s t-test unless otherwise noted. A *p*-value <0.05 was considered statistically significant. Where results from representative experiments including micrographs or NIR-nCLE images, the experiments were repeatedly independently at least three times. The sensitivity, specificity, positive predictive value, negative predictive value, accuracy, and the IOR were calculated using the standard definitions and software from the SPSS statistical package V.25.0 (IBM Corporation). Statistical analysis was conducted using Graphpad Prism (Version 8.4.1, GraphPad Software, La Jolla, CA, US), Microsoft Excel 2016, SPSS statistical package V 25.0 (IBM Corporation) and image analysis was conducted using Matlab (version 2020b and 2021a, MathWorks, Natick, MA), ImageJ version 1.52k and Image Studio version 5.2.

### Reporting summary

Further information on research design is available in the Nature Research Reporting Summary linked to this article.

## Supplementary information

Supplementary Information

Reporting Summary

## Data Availability

The data associated with this study are presented in the paper, supplementary information and Source Data file. NIR-nCLE imaging sequences from this study are available upon request to the Corresponding Author. Source data are provided with this paper.

## Source data

Source Data
