# Supplementary material for: Targeted detection of cancer at the cellular level during biopsy by near-infrared confocal laser endomicroscopy
Source: Nat Commun. 2022 May 17;13:2711. doi: 10.1038/s41467-022-30265-z (PMC9114105; doi:10.1038/s41467-022-30265-z)
Supplement: Supplementary file 1 — Supplementary Information [file 41467_2022_30265_MOESM1_ESM.pdf]

Supplementary Information

Targeted Detection of Cancer at the Cellular Level During Biopsy by Near-Infrared Confocal Laser Endomicroscopy

Gregory T. Kennedy, MD[1], Feredun S. Azari, MD[1], Elizabeth Bernstein, BA[1], Bilal Nadeem, BA[1], Ashley Chang, BA[1], Alix Segil, BA[1], Sean Carlin, PhD[2], Neil T. Sullivan, PhD[1], Emmanuel Encarnado, BA[1], Charuhas Desphande, MD[3], Sumith Kularatne, PhD[4], Pravin Gagare, PhD[4], Mini Thomas, PhD[4], John C. Kucharczuk, MD[1], Gaetan Christien, MSc[5], Francois Lacombe, PhD[5], Kaela Leonard, PhD[5], Philip S. Low, PhD[6], Aline Criton, PhD[5], Sunil Singhal, MD[1]

[1]Department of Surgery, University of Pennsylvania School of Medicine, Philadelphia, PA
[2]Department of Radiology, University of Pennsylvania School of Medicine, Philadelphia, PA
[3]Department of Pathology, University of Pennsylvania School of Medicine, Philadelphia, PA
[4]On Target Laboratories, West Lafayette, IN
[5]Mauna Kea Technologies, Paris, France
[6]Department of Chemistry, Purdue University, West Lafayette, IN

Corresponding Author:
Sunil Singhal, MD
Department of Surgery
University of Pennsylvania Perelman School of Medicine
3400 Spruce Street, 6 White Building
Philadelphia, PA 19104
sunil.singhal@pennmedicine.upenn.edu

Inventory of Supplementary Information

Supplementary Figure 1	p. 3
Supplementary Figure 2	p. 4
Supplementary Figure 3	p. 5
Supplementary Figure 4	p. 6
Supplementary Figure 5	p. 7

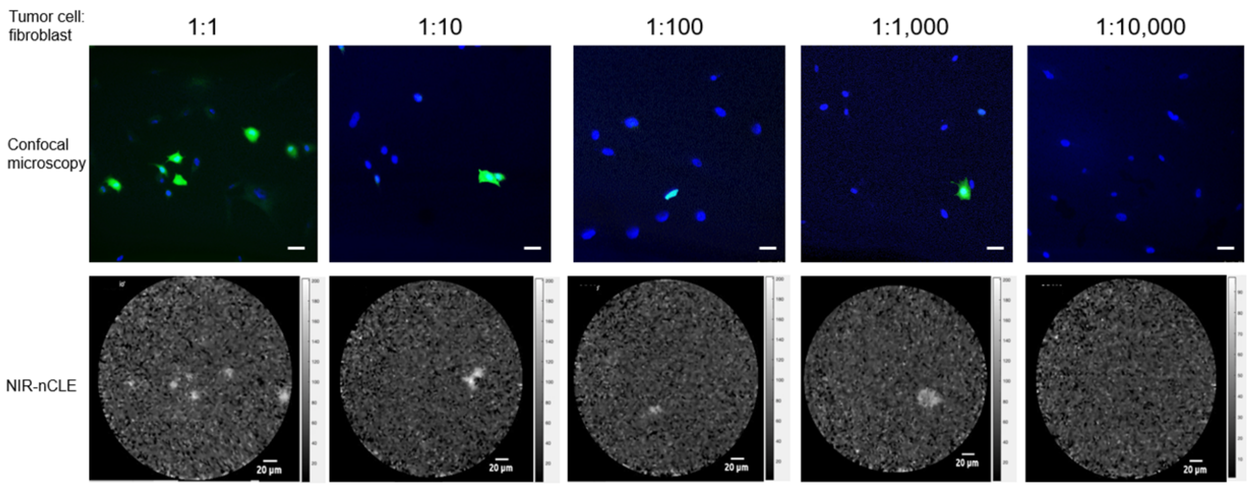

Supplementary Figure 1. NIR-nCLE differentiates benign from malignant cells. Representative paired confocal microscopy images and NIR-nCLE images of A549-GFP cells (human lung adenocarcinoma) co-cultured with HD28 cells (human normal lung fibroblast) at various ratios. In the confocal microscopy images, fluorescence at GFP wavelength (510 nm) is overlaid on DAPI staining images. The lower limit of detection of NIR-nCLE for a single malignant cell was when tumor and normal cells were co-cultured at a ratio of 1:1000. Scale bars represent 20 µm.

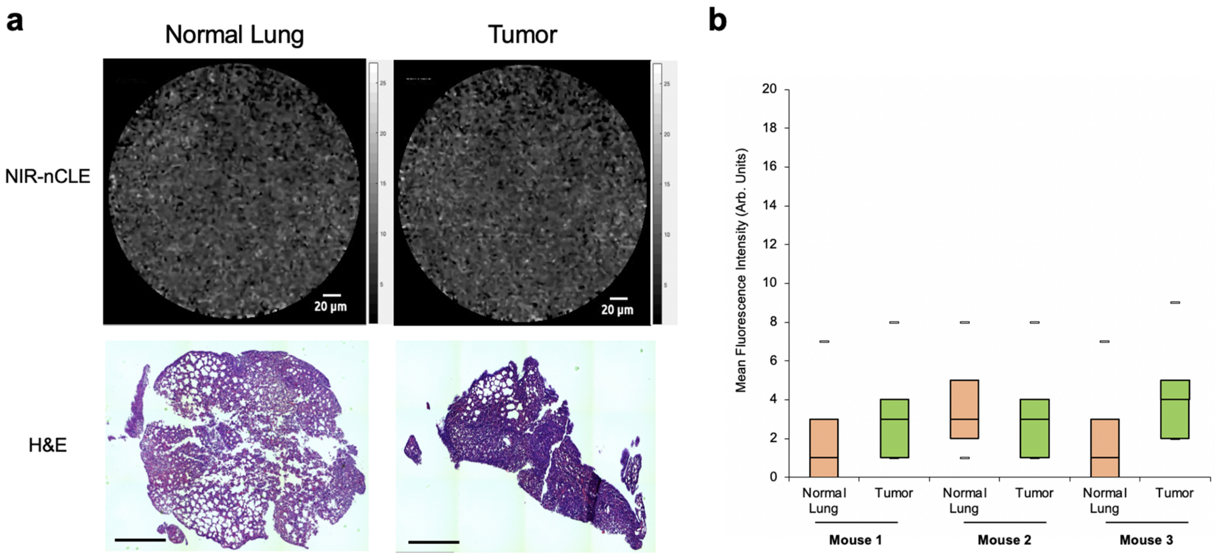

Supplementary Figure 2. NIR-nCLE of normal lung and tumor tissue in mice bearing orthotopic pulmonary tumors and infused with PBS vehicle as a negative control. **a.** Representative NIR-nCLE images and paired hematoxylin and eosin-stained biopsy slides of normal lung and tumor, respectively. Scale bars represent 5 mm. **b.** Mean fluorescence intensity (MFI) of NIR-nCLE sequences taken during biopsy of normal lung and tumor tissue in mice infused with PBS 24 hours prior to biopsy. Minima, centre, and maxima of boxes represent 25th percentile, mean, and 75th percentile of MFI obtained for the entire imaging sequence. (n = 114, 115, and 117 frames for normal lung biopsies for Mice 1, 2, and 3, respectively; n = 117, 117, and 116 frames for tumor biopsies in Mice 1, 2, and 3, respectively). Whiskers represent the 5th and 95th percentile of MFI over the same sequence. There were no significant differences in MFI between normal lung and tumor tissue.

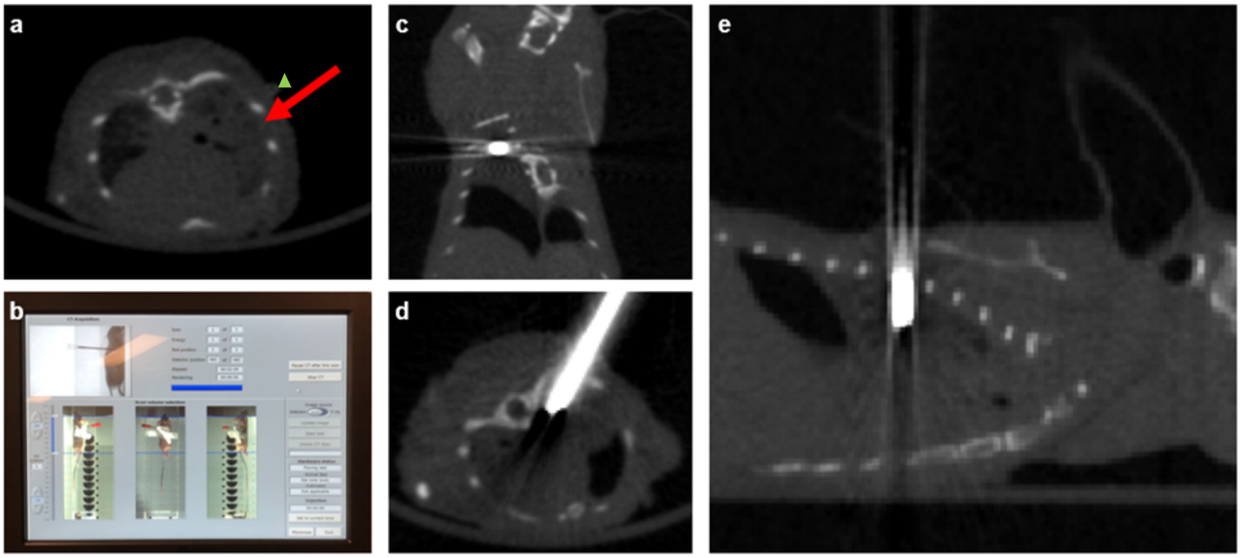

Supplementary Figure 3. Overview of transthoracic biopsy model in nude athymic mice bearing human non-small cell lung cancer orthotopic xenografts. a. Pre-biopsy CT scans were taken in mice to assess the location of the tumor in relation to an overlying radiopaque marker on the skin surface. Red arrow marks tumor and green arrowhead marks location of radiopaque surface marker. **b.** Screen interface of the micro-CT scanner, showing fluoroscopic scout films of the needle location. **c., d., and e.** Coronal, axial, and sagittal reconstructions of the CT scan were generated to confirm needle location before NIR-nCLE imaging and tissue biopsy.

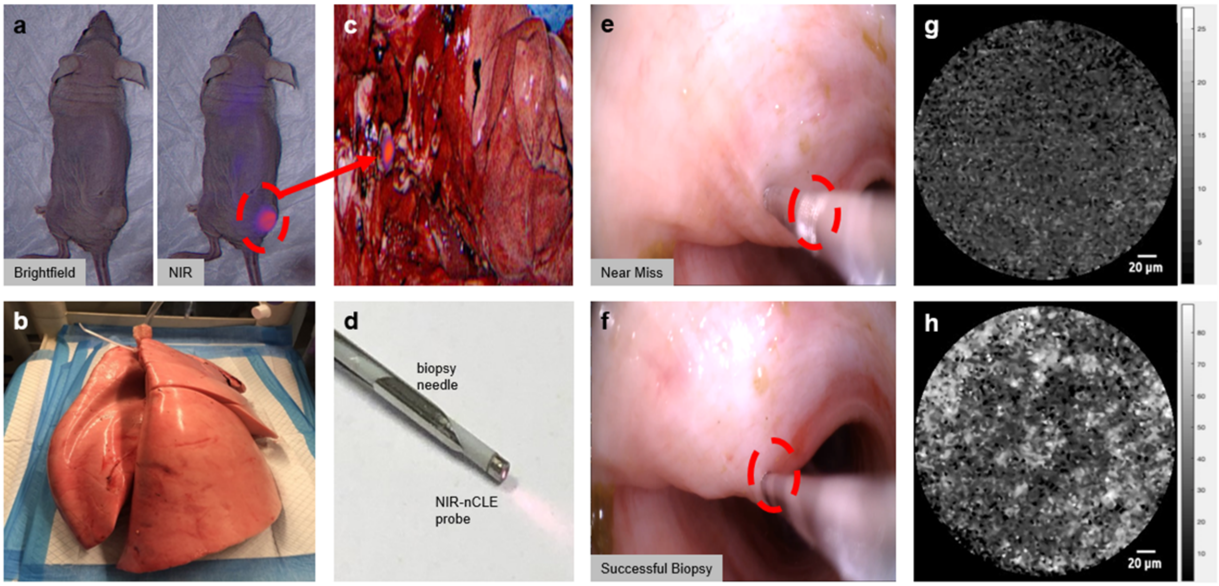

Supplementary Figure 4. Overview of the bronchoscopic biopsy model in cadaveric caprine lungs. **a.** Brightfield and NIR images of a nude, athymic mouse bearing a human NSCLC flank xenograft 24 hours after administration of 2 mg/kg pafolacianine. **b.** Cadaveric caprine lungs intubated with an endotracheal tube and inflated. **c.** NIR images of caprine lungs after peri-bronchial implantation of the resected murine flank xenograft. **d.** Image of NIR-nCLE probe within the lumen of a flexible 19-gauge bronchoscopic biopsy needle. **e. and f.** Bronchoscopic images of a representative near-miss biopsy of normal lung and successful tumor biopsy, respectively. Red circle denotes the tumor indenting the bronchial wall. **g. and h.** NIR-nCLE images obtained at the time of near-miss or successful biopsy, respectively.

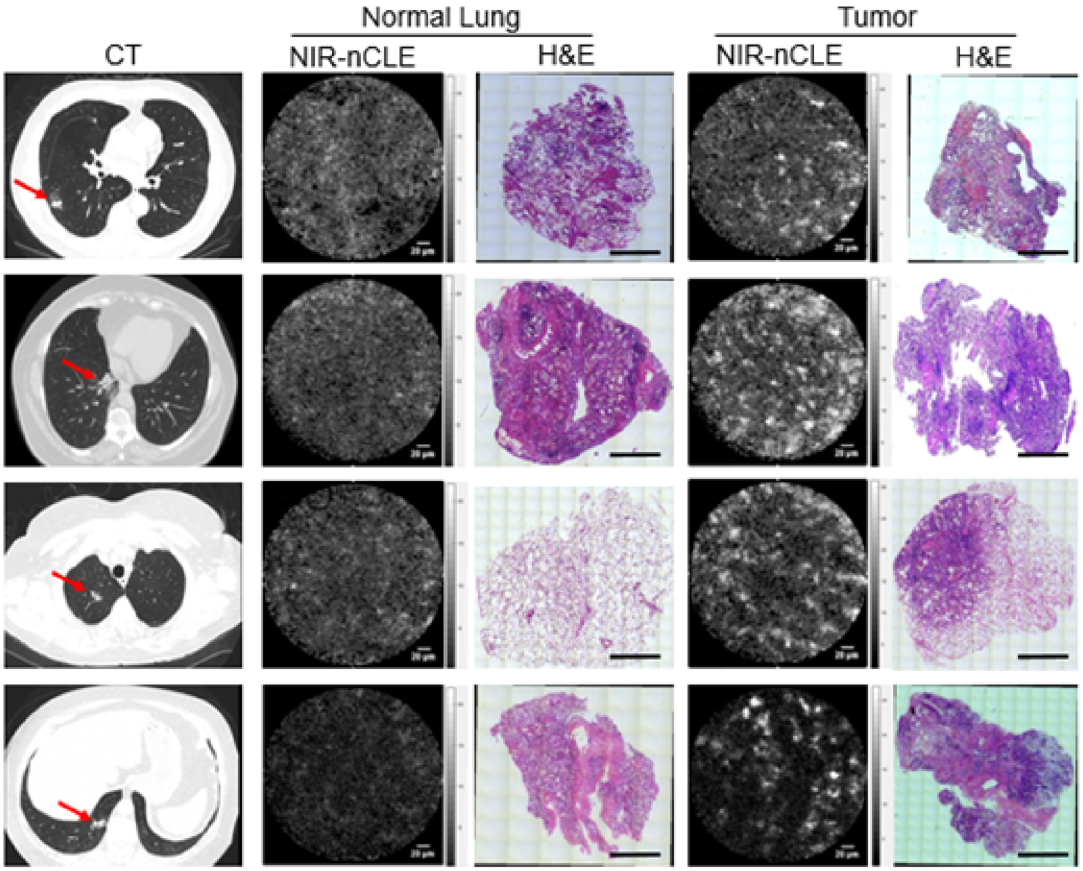

Supplementary Figure 5. NIR-nCLE distinguishes tumor from normal tissue in human pulmonary tumors. In the leftmost column are representative preoperative chest CT scans showing the four part-solid ground glass opacity lesions not presented in Figure 5. Red arrows mark lesion location. Adjacent to each CT scan are representative NIR-nCLE images taken from normal lung and tumor tissue with paired H&E slides of the biopsy specimens obtained from the same location. Scale bars represent 5 mm.
