# Supplementary material for: Targeted detection of cancer at the cellular level during biopsy by near-infrared confocal laser endomicroscopy
Source: Nat Commun. 2022 May 17;13:2711. doi: 10.1038/s41467-022-30265-z (PMC9114105; doi:10.1038/s41467-022-30265-z)
Supplement: Supplementary file 2 — Reporting Summary [file 41467_2022_30265_MOESM2_ESM.pdf]

nature portfolio

Corresponding author(s): Gregory Kennedy, MD
Sunil Singhal, MD

Last updated by author(s): April 11, 2022

Reporting Summary

Nature Portfolio wishes to improve the reproducibility of the work that we publish. This form provides structure for consistency and transparency in reporting. For further information on Nature Portfolio policies, see our Editorial Policies and the Editorial Policy Checklist.

Statistics

For all statistical analyses, confirm that the following items are present in the figure legend, table legend, main text, or Methods section.

n/a	Confirmed	
☐	☒	The exact sample size (*n*) for each experimental group/condition, given as a discrete number and unit of measurement
☐	☒	A statement on whether measurements were taken from distinct samples or whether the same sample was measured repeatedly
☐	☒	The statistical test(s) used AND whether they are one- or two-sided<br>*Only common tests should be described solely by name; describe more complex techniques in the Methods section.*
☐	☒	A description of all covariates tested
☒	☐	A description of any assumptions or corrections, such as tests of normality and adjustment for multiple comparisons
☐	☒	A full description of the statistical parameters including central tendency (e.g. means) or other basic estimates (e.g. regression coefficient) AND variation (e.g. standard deviation) or associated estimates of uncertainty (e.g. confidence intervals)
☐	☒	For null hypothesis testing, the test statistic (e.g. *F*, *t*, *r*) with confidence intervals, effect sizes, degrees of freedom and *P* value noted<br>*Give P values as exact values whenever suitable.*
☒	☐	For Bayesian analysis, information on the choice of priors and Markov chain Monte Carlo settings
☒	☐	For hierarchical and complex designs, identification of the appropriate level for tests and full reporting of outcomes
☒	☐	Estimates of effect sizes (e.g. Cohen's *d*, Pearson's *r*), indicating how they were calculated

Our web collection on statistics for biologists contains articles on many of the points above.

Software and code

Policy information about availability of computer code

Data collection	The macroscopic fluorescence images of primary tumor specimens were collected by using Image Studio version 5.2; The microscopic fluorescence images were acquired by using Leica Application Suite X 3.0.2.16120.
Data analysis	Statistical analysis: Graphpad Prism (Version 8.4.1, GraphPad Software, La Jolla, CA, US), Microsoft Excel 2016, SPSS statistical package V 25.0 (IBM Corporation); Image analysis: Matlab (version 2020b and 2021a, MathWorks, Natick, MA); imageJ version 1.52k; Image Studio version 5.2

For manuscripts utilizing custom algorithms or software that are central to the research but not yet described in published literature, software must be made available to editors and reviewers. We strongly encourage code deposition in a community repository (e.g. GitHub). See the Nature Portfolio guidelines for submitting code & software for further information.

Data

Policy information about availability of data

All manuscripts must include a data availability statement. This statement should provide the following information, where applicable:

- Accession codes, unique identifiers, or web links for publicly available datasets
- A description of any restrictions on data availability
- For clinical datasets or third party data, please ensure that the statement adheres to our policy

The source data underlying Figs 1e, 2b, 2d, 3a, 3l, 4e, 5d, and S2b are provided as a Source Data file. All other data that support the findings of this study are available from the corresponding author upon request.

Field-specific reporting

Please select the one below that is the best fit for your research. If you are not sure, read the appropriate sections before making your selection.

☒ Life sciences ☐ Behavioural & social sciences ☐ Ecological, evolutionary & environmental sciences

For a reference copy of the document with all sections, see nature.com/documents/nr-reporting-summary-flat.pdf

Life sciences study design

All studies must disclose on these points even when the disclosure is negative.

Sample size	No sample-size calculation was performed. For studies in cell lines and preclinical models, sample sizes were chosen according to previous experimental experience in our laboratory and in other preclinical evaluations of CLE technology (see references below). For the validation study in human resection specimens, the sample size was chosen based upon World Molecular Imaging Society guidelines in conjunction with our prior institutional experience with intraoperative imaging clinical study design.<br><br>References:<br><br>Predina JD et al. Molecular Therapy, 2018; 26:390-403.<br><br>Kennedy GT et al. Molecular Cancer Therapeutics 2022; 21: 546-554.<br><br>Samarasena JB et al. J Gastroenterol Hepatol 2016; 31:802-7.<br><br>Becker V et al. Gastrointest Endosc 2010:71:1260-6.<br><br>Tummers WS et al. Cancer Res. 2017;77:2197–206
Data exclusions	No data were excluded from the analysis.
Replication	Preclinical data are presented as means and standard deviations of at least three independent experiments. All attempts at replication were successful.
Randomization	Mice were allocated randomly to each experimental group. In studies assessing the diagnostic accuracy of NIR-nCLE, all imaging sequences were randomized by a study administrator (Dr. Leonard) blinded to the diagnosis associated with each imaging sequence.
Blinding	In studies assessing the diagnostic accuracy of NIR-nCLE, all raters were blinded to the histopathologic diagnosis associated with individual NIR-nCLE sequences. Raters were not involved in the initial acquisition of NIR-nCLE sequences. Investigators were also blinded to group allocation during data collection and analysis of the additional preclinical experiments described in this manuscript.

Reporting for specific materials, systems and methods

We require information from authors about some types of materials, experimental systems and methods used in many studies. Here, indicate whether each material, system or method listed is relevant to your study. If you are not sure if a list item applies to your research, read the appropriate section before selecting a response.

Materials & experimental systems

n/a	Involved in the study	
☒	☐	Antibodies
☐	☒	Eukaryotic cell lines
☒	☐	Palaeontology and archaeology
☐	☒	Animals and other organisms
☐	☒	Human research participants
☐	☒	Clinical data
☒	☐	Dual use research of concern

Methods

n/a	Involved in the study	
☒	☐	ChIP-seq
☒	☐	Flow cytometry
☒	☐	MRI-based neuroimaging

Eukaryotic cell lines

Policy information about cell lines

Cell line source(s)	A549 human lung adenocarcinoma cells, H1264 human squamous cell lung cancer cells, and KB human cervical carcinoma cells were obtained from the American Type Culture Collection (ATCC). Luciferase-transfected TC1 mouse adenocarcinoma cells and HD28 human lung fibroblast cells were obtained from the laboratory of Dr. Steven Albelda (University of

	Pennsylvania). The original source of these cell lines was the ATCC.
Authentication	Each cell line used was morphologically confirmed according to the information provided by culture collections.
Mycoplasma contamination	All the cell lines presented in this study were tested for mycoplasma contamination and they were free of mycoplasma contamination.
Commonly misidentified lines (See ICLAC register)	No commonly misidentified cell lines were used.

Animals and other organisms

Policy information about studies involving animals; ARRIVE guidelines recommended for reporting animal research

Laboratory animals	Female, 6-8 week old, athymic nude mice were purchased from Charles River Laboratories (Wilmington, MA).<br><br>All mice were housed in the vivarium at the University of Pennsylvania, where they were maintained on a normal mouse chow diet and a 12 h/12 h light (<10 lux)/dark cycle. The temperature ranged from 75 to 76 °F, and the humidity from 30 to 40%.
Wild animals	No wild animals were used in the study.
Field-collected samples	No field-collected samples were used in the study.
Ethics oversight	Ethical approval for all animal studies was obtained from the Institutional Animal Care and Use Committee of the University of Pennsylvania (IACUC protocol numbers 803344 and 806483).

Note that full information on the approval of the study protocol must also be provided in the manuscript.

Human research participants

Policy information about studies involving human research participants

Population characteristics	Patients, M/F, >19 years of age, with a sub-solid pulmonary nodule (ground glass opacity) radiographically suspicious for malignancy scheduled to undergo surgical resection were eligible to participate in the study. A total of 5 patients were enrolled in the pilot study.<br><br>Characteristics of the patients are as follows: 4/5 patients were female. The average age was 67 years (range 53-82) and all patients had a fine-cut CT scan indicating a sub-solid pulmonary nodule suspicious for malignancy and were scheduled to undergo surgical resection of the primary lesion. Final histopathologic diagnoses were invasive adenocarcinomas (n=3, 60%), one minimally invasive adenocarcinoma, and one adenocarcinoma in situ.
Recruitment	Patients were recruited from the thoracic surgery practice at Penn. All patients were evaluated by Dr. Singhal and a medical oncologist to ensure that the patient met all eligibility criteria. Patients were initially approached by the protocol director, Dr. Singhal.<br><br>Written Informed Consent and HIPAA Authorization were obtained after the Informed Consent was reviewed and the study was fully explained to the patient including potential risks and discomforts – this was done by the clinical trial coordinators (Ms. Azra Din and Ms. Isvita Marfatia). Full disclosure of the details and the investigational nature of the proposed protocols was provided by both the protocol director, Dr. Singhal and by the clinical trial coordinators.<br><br>Bias: To the best of our knowledge, self-selection bias did not occur.
Ethics oversight	The protocol adhered to regulations to provide protection for human subjects in clinical investigations described by the general requirements for informed consent. The IRB of the University of Pennsylvania provided oversight for the trial and trial safety (IRB number 822153).

Note that full information on the approval of the study protocol must also be provided in the manuscript.

Clinical data

Policy information about clinical studies

All manuscripts should comply with the ICMJE guidelines for publication of clinical research and a completed CONSORT checklist must be included with all submissions.

Clinical trial registration	NCT02602119
Study protocol	The study protocol (Penn IRB: 822153) is available from the corresponding author upon reasonable request.
Data collection	Enrolled patients underwent surgery between 4/2021-7/2021. On the day of surgery, fluorescence imaging data was gathered in the operating theatre. Subsequently, fluorescence imaging data was gathered during the pathological processing steps; this data was collected in the Singhal Laboratory at the University of Pennsylvania. Data analysis, including fluorescence imaging data analysis, immunohistochemistry and statistical analysis was performed between 8/2021 and 11/2021 in the Singhal Laboratory at the University of Pennsylvania.
Outcomes	Primary outcome: To investigate whether NIR-nCLE can provide clear images of pafolacianine labeled fluorescent cancer cells with

Outcomes

appropriate resolution in lung tumors in an ex-vivo setting.

Assessed as follows:

Study participants will receive intravenous pafolacianine (0.025 mg/kg) on the day prior to resection per an established protocol for fluorescent guided surgery. During the surgical procedures, lung nodules will be resected with a curative intent. Excised tissue specimens were subjected to ex-vivo NIR-nCLE examination immediately after resection prior to classical pathological, immunofluorescent (IF), and immunohistochemistry (IHC) analysis. Results from NIR-nCLE imaging analysis will be compared to histology on same or similar locations of the tissue sample. Excised tissue from patients without being infused with pafolacianine will be used as negative controls.

Processed images and video will be displayed in real time on the NIR-nCLE system monitor and recorded while moving the probe within the tissue sample. The overall impression of the quality of images (scale 1-5), whether clear images of individual cells (Yes/No) and the mean fluorescence intensity (MFI) and signal to background ratio (SBR) (after software calculation) will be recorded at each contact point prior to biopsy.

Secondary outcome: To investigate the sensitivity and specificity of diagnosing pulmonary lesions using NIR-nCLE.

Assessed as follows:

For each NIR-nCLE video sequence, five blinded raters will evaluate independently the presence or absence of predefined NIR-nCLE criteria where the presence of at least one NIR-nCLE criterion will mean that this criterion is present for this NIR-nCLE contact point. The final CLE criterion presence or absence will be determined after final review among the raters. If there is no consensus is reached for a criterion on a contact position, rater's individual results will be kept. For each NIR-nCLE video sequence analysis, the ratings will be unblinded and the diagnostic performance will be compared to definitive pathological results as the reference (c.f. Standards for Reporting of Diagnostic Accuracy (STARD) guidelines). The interobserver variability will be calculated using multirater Fleiss's kappa statistic with the following classification: poor 0–0.2, fair 0.21–0.4, moderate 0.41–0.6, substantial 0.61–0.8, and excellent 0.81–1 for each criterion.
